# Ecotoxicity Evaluation of Fire-Extinguishing Water from Large-Scale Battery and Battery Electric Vehicle Fire Tests

**DOI:** 10.1021/acs.est.2c08581

**Published:** 2023-03-13

**Authors:** Maria Quant, Ola Willstrand, Tove Mallin, Jonna Hynynen

**Affiliations:** Department of Fire and Safety, RISE Research Institutes of Sweden, Brinellgatan 4, 501 15 Borås, Sweden

**Keywords:** battery electric vehicle, lithium-ion battery, fire test, extinguishing water, ecotoxicity

## Abstract

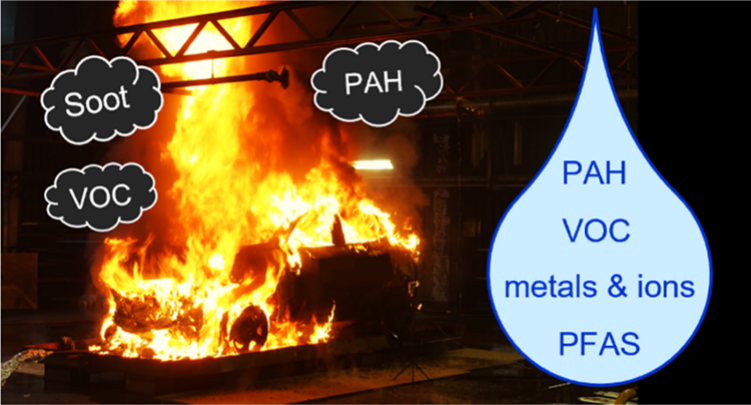

Electrified transport has multiple benefits but has also raised some concerns, for example, the flammable formulations used in lithium-ion batteries. Fires in traction batteries can be difficult to extinguish because the battery cells are well protected and hard to reach. To control the fire, firefighters must prolong the application of extinguishing media. In this work, extinguishing water from three vehicles and one battery pack fire test were analyzed for inorganic and organic pollutants, including particle-bound polycyclic aromatic hydrocarbons and soot content. Additionally, the acute toxicity of the collected extinguishing water on three aquatic species was determined. The vehicles used in the fire tests were both conventional petrol-fueled and battery electric. For all of the tests, the analysis of the extinguishing water showed high toxicity toward the tested aquatic species. Several metals and ions were found in concentrations above the corresponding surface water guideline values. Per- and polyfluoroalkyl substances were detected in concentrations ranging between 200 and 1400 ng L^–1^. Flushing the battery increased the concentration of per- and polyfluoroalkyl substances to 4700 ng L^–1^. Extinguishing water from the battery electric vehicle and the battery pack contained a higher concentration of nickel, cobalt, lithium, manganese, and fluoride compared with the water samples analyzed from the conventional vehicle.

## Abstract 2

Minimal research exists on pollutants from vehicle fires. This study reports differences in the polluting species found for fires in lithium-ion battery electric vehicles and internal combustion engine vehicles.

## Introduction

Combustion products from fires contribute to contamination of the atmospheric, aquatic, and terrestrial environments. The degree of seriousness will depend on the material combusted, the size and duration of a fire, ventilation conditions, and even the firefighting tactics used upon suppression.^1,2^ During a vehicle fire, toxic gases containing volatile organic compounds (VOCs),^3,4^ polycyclic aromatic hydrocarbons (PAHs),^5−9^ hydrogen halides (HX),^10^ soot particulates,^11,12^ etc. are released.^10^ Single-vehicle fires can be considered as relatively small events compared to, for example, fires in buildings. However, in Sweden, a total of ∼5000 vehicle fires occur each year,^13^ resulting in a local environmental impact. A large number of vehicle fires each year may therefore pose a risk of significant cumulative emission, especially for the more persistent compounds.

Another negative contribution to the environment from vehicle fires is the resulting fire-extinguishing water runoff.^14−16^ Water or other extinguishing media used during a firefighting operation may introduce large amounts of polluted water into the environment, as particulate matter tends to be “washed out” from the smoke-plume upon application of an extinguishing agent. In work by Lönnermark and Blomqvist,^15^ combustion gases and extinguishing water from vehicle fires were investigated. Results showed that the extinguishing water contained elevated levels of organic compounds as well as metals such as lead, copper, zinc, and antimony.

As a part of reducing the use of fossil fuels, internal combustion engine vehicles (ICEVs) are being replaced by battery electric vehicles (BEVs). Today, BEVs are powered by lithium-ion batteries (LIBs). LIBs contain materials, especially metals^10^ and fluoride-containing compounds,^17^ that are not found in ICEVs. Currently, the anode-active material used in commercial applications is usually graphite,^18^ while the cathode-active material comes in a variety of lithiated materials.^19^ The electrolyte is typically composed of an organic carbonate-based solvent (for example, ethylene carbonate and dimethyl carbonate) that is mixed with a lithium salt such as LiPF_6_, LiClO_4_, or LiBF_4_.^20^

Thermal runaway, the more severe type of battery failure, can be induced by mechanical, electric, or thermal abuse.^21,22^ Thermal runaway is often attributed to the failure of the separator/interphase materials, resulting in an internal short circuit.^23^ When separator/interphase materials are damaged, exothermic chemical reactions are initiated between the cathode, anode, and electrolyte. These exothermic reactions are followed by an increase in pressure, which can eventually lead to cell rupture and the release of toxic and flammable gases.^24−26^ Some cell chemistries also release oxygen when exposed to high temperatures,^21^ which can lead to auto-ignition of the released gases. The state of charge (SOC) of the battery does not influence the total energy that can be released from the battery, but it contributes to the activation energy of the heat-release processes. The chemical energy stored in the materials is the main source of thermal energy in batteries.^25,27^

Fires in BEVs, where the traction battery is involved in the fire, are more difficult to extinguish than fires in ICEVs because the battery cells are well protected in the vehicle chassis. Since the battery cells are difficult to reach, large quantities of water or other extinguishing media are generally required.^28^

Until now, only a handful of large-scale fire tests on BEVs have been performed.^10,29−33^ Results from these studies show that a typical vehicle fire lasts for 60–90 min and has a peak heat-release rate (HRR) in the range of 1.5–8 MW^15^ and an average total heat release (THR) of ∼5.9 GJ.^10^ The total available chemical energy in a vehicle varies and depends on the type, size, and material of the vehicle. For example, plastics used for, e.g., seating/upholstery correspond to an average of ∼20%^34^ of the total weight of a passenger vehicle and will considerably affect the combustion behavior.^32^ In work by Willstrand et al.,^10^ it was reported that an increased concentration of gaseous hydrogen fluoride, nickel, cobalt, lithium, and manganese was found in the combustion gases from BEV fires compared to ICEV fires.

The focus of previous large-scale fire tests on BEVs has been on the fire scenarios and analysis of combustion gases. In this work, acute toxicity tests of fire-extinguishing water from large-scale vehicle fire tests were performed. Three large-scale vehicle fire tests and one LIB fire test were conducted. To the best of our knowledge, this is the first time that chemical analysis and acute toxicity tests have been performed on fire-extinguishing water resulting from a large-scale BEV fire.

## Materials and Methods

### Test Setup and Test Objects

Three large-scale vehicle fire tests and one battery fire test were performed in a fire hall equipped with a calorimeter hood to enable the collection and analysis of smoke and gas emissions. A schematic figure of the test hall is presented in Supporting Information (SI) Figure S1. Advanced flue gas reduction and water purification systems are linked to the fire hall to minimize exhausts to the environment upon testing. The fires were suppressed by an overhead sprinkler system, and the sprinkler system utilized tap water as a suppressant. Detailed information about the sprinkler system can be found in SI Section S1.2. The extinguishing water was collected by a tray-pump system, where the extinguishingwater was pumped to an adjacent hall for sampling (SI Figure S2).

In total, four tests were performed, and the tested vehicles were one BEV, one ICEV, and one BEV, where the battery pack was removed to provide a reference test. Additionally, a test was performed using the battery pack that was removed from the BEV for the reference test. The BEV and battery pack had a 50 kWh, 18 module, 216 cell, lithium–nickel–manganese–cobalt oxides (NMC)-type battery with a SOC of 90%. All vehicles and the battery were new and unused. Vehicles used were manufactured in 2021 by the same manufacturer and of the same model and size, which enabled a good comparison between the powertrains.

For safety reasons, some modifications of the vehicles were necessary. These modifications included puncturing the tires and disabling dampers and suspensions. In addition, airbags were not active in the test vehicles. The plastic shield covering the underside of the vehicles was removed to accommodate the propane gas burner used to initiate the fire. The propane gas burner had a defined power output of 30 kW. As a safety measure, the propane gas burner was kept active throughout the duration of the tests to ignite potential flammable gases produced during the tests, minimizing the risk of gas explosions.^24^

For the BEV without a battery and for the ICEV, the propane gas burner was placed below the engine compartment of the vehicle. For safety reasons, the fuel tank in the ICEV test was filled with ∼20 L of petrol (half-full tank). The remaining petrol (20 L) was poured into a tray (1.0 × 1.1 × 0.1 m) below the tank to mimic a fuel leak and a resulting pool fire. For the BEV and the battery pack, the burner was located below the rear of the battery pack to ensure the involvement of the battery in the fire as early as possible.

The battery pack was shielded to reduce direct water exposure from the sprinkler system to the battery casing, corresponding to the protection of the chassis. In all tests, a large steel tray (5.0 × 2.0 × 0.15 m), equipped with a water outlet connected to a pump, was positioned under the test object to collect the applied water from the sprinkler system. Smoke and gases generated during the tests were collected in a hood and exhausted through a duct, and the distance between the duct and the ground was 8 m. The flow rate in the duct for all three tests was ∼25 m^3^ s^–1^.

### Fire Scenario and Heat-Release Rate

Visual observations along with temperature and HRR measurements were carried out to monitor the fire scenario. To calculate the HRR, an industrial calorimeter was used. The calorimeter collects combustion products in the hood before extraction through the exhaust duct. A set of guide plates and a sufficient length of the duct (∼30 m) were used to reduce the turbulence at the sampling point. Upon activation of the sprinkler system, the smoke plume will spread in the fire hall and the uptake in the hood becomes slightly lower compared to a free burning test. Therefore, the HRR values reported for the sprinklered tests are expected to include a larger measurement uncertainty compared with the free burning test (reference test). Equations used for the calculation of HRR can be found in SI Section S1.3. Details about the temperature measurements can be found in SI Section 2.2 and Figure S3.

### Filter Sampling

Sampling of soot particles in the exhaust duct was performed by isokinetic sampling on quartz filters. The flow rate was set to 50 L min^–1^ at the sampling point, and the sampled gas flow was divided between two identical filters. Filters were dried at ambient temperature before analysis. For analysis of PAHs, the filter was extracted using toluene in an ultrasonic bath for 30 min and extracts were analyzed using gas chromatography–mass spectrometry (GC–MS); 16 external standards were used (SI Table S1).

### Sampling and Analysis of Extinguishing Water

The extinguishing water was collected in a customized steel tray (5.0 × 2.0 × 0.015 m) that was placed beneath the test object. The collected water in the steel tray was drained through two openings to an adjacent pump-tray (0.15 m^3^). The pump-tray was located beneath the large tray, and the collected water was pumped to an adjacent test hall at a flow of ∼2 L min^–1^ using a heavy-duty pump.

The pump delivered ∼3600 L of water during 30 min, i.e., roughly a third of the total amount of water delivered by the sprinkler system during the test. The remaining water was collected and cleaned using the ordinary water purification system connected to the fire hall. The collection of water started when the sprinkler system was started. One liter of water was collected into a clean flask each minute during the time when the sprinklers were active. At the end of each test, the vehicles and battery were flushed for 5 min, and 0.5 L of the water left in the tray was collected for analysis. In total, five water samples from each sprinklered test were collected: (1) 0–10 min, (2) 11–20 min, (3) 21–30 min, (4) 0–30 min (equal mixture of samples 1–3), and (5) sample collected from the tray at the end of the test.

The whole test setup (including tray, hoses, pump, etc.) was flushed with clean tap water for a minimum of 10 min between each test. The water used for flushing (at *t* = 10 min) was collected as a blank sample (background reference sample).

For analysis of the inorganic species, water samples were filtered (0.45 μm) before being determined by Inductively Couples Plasma Mass Spectroscopy (ICP-MS) and ICP Optical Emission Spectrometry (ICP-OES). Water-soluble contents of fluoride, chloride, and bromide were analyzed using ion chromatography (IC) with a conductivity detector.

For analysis of VOCs, water samples (100 mL) were extracted with dichloromethane (DCM) after the addition of internal standard bis(2-ethylhexyl) phthalate-d4 (DEHP-d, 10 μg, 1 mg ml^–1^) in DCM. The extracts were evaporated to 0.2–0.5 mL, followed by analysis using GC–MS with an Agilent JW Scientific DB-5MS column (30 m x 0.250 mm, 0.25 μm film thickness). Detected compounds were identified using the National Institute of Standards and Technology (NIST) library of mass spectra, and the concentrations were determined in equivalents of the internal standard DEHP-*d*_4_. The suitability of the VOC screening method was validated by analysis of an external mixture of octamethylcyclotetrasiloxane, bis(2-ethylhexyl) terephthalate, butylated hydroxytoluene, DEHP, bisphenol A, and Irganox 1076 at concentrations corresponding to 10–100 μg L^–1^ in water samples. Six concentrations of the standard in addition to blank analysis, each injected in duplicate, were used for the assessment of linearity and report limit. The limit of quantification corresponds to 10 μg L^–1^ in the water samples. Of each sample, 10 mL was analyzed by headspace GC–MS after the addition of an internal standard benzene-*d*_6_ (0.10 mg L^–1^ in 10 μL water) and heating at 95 °C for 30 min. The compounds detected were again identified using the NIST library of mass spectra, and the concentrations were determined in equivalents of the internal standard benzene-*d*_6_. The PAH concentrations of the DCM extracts were determined using GC–MS. Naphthalene-*d*_8_, chrysene-*d*_12_, and benzo[*a*]pyrene-*d*_12_ were used as internal standards (1.0 mg L^–1^ in 10 μL DCM), and 16 external standards were used (SI Table S1).

Per- and polyfluoroalkyl substances (PFAS) were analyzed using liquid chromatography tandem mass spectrometry (LC/MS/MS). The LC/MS/MS instruments were a Waters Acquity UPLC I-class LC-system and a Waters Xevo TQ-XS mass spectrometer. A Waters Acquity UPLC BEH C18, 2.1 mm × 100 mm, 1.7 μm column was used for chromatographic separation of the analytes. Sample preparation and analysis were made according to ASTM D7979–19 “Standard Test Method for Determination of Per- and Polyfluoroalkyl Substances in Water, Sludge, Influent, Effluent, and wastewater by Liquid Chromatography Tandem Mass Spectrometry (LC/MS/MS)”. The targeted PFAS and the limit of quantification can be found in SI Table S10.

### Acute Toxicity Tests of Extinguishing Water

Acute toxicity tests were performed by an external laboratory, Toxicon AB. Extinguishing water collected from the pumped water, 0–30 min sample (ICEV, BEV, and battery test), was frozen and sent to Toxicon AB for analysis. Measurements of pH, salinity, and conductivity were conducted before characterization.

Microtox analysis was performed on all samples according to SS-EN ISO 11348-3:2008 “Determination of the inhibitory effect of water samples on the light emission of *Vibrio fischeri”.* The control sample was tested against phenol and had a half-maximal effective concentration (EC_50_) for 5 min exposure of 17.1 mg phenol L^–1^, approved range 13–26 mg L^–1^. Salt content and pH were adjusted to 20 ppt (with NaCl) and 6.8–7.2 (with HCl or NaOH), respectively, before testing. The detailed test method, test concentrations, and calculations for the inhibitory effect on luminescence are found in SI Section 1.5.1.

Growth inhibition rate (E_r_C_10_ and E_r_C_50_) of *Pseudokirchneriella subcapitata* (Green algae) was assessed for samples from the ICEV and BEV according to SS-EN ISO 8692:2012 “Fresh water algal growth inhibition test with unicellular green algae”. The control sample was tested against K_2_Cr_2_O_7_ and had a E_r_C_50_ of 1.4 mg K_2_Cr_2_O_7_ L^–1^, approved range 0.9–1.5 mg L^–1^. For the ICEV water sample, the pH was adjusted to 8.1 ± 0.2 (with NaOH) before testing. Exposure concentrations for test 2 (ICEV) was: 0.18, 0.35, 0.70, 1.4, 2.8, and 5.6% vol/vol and for test 3 (BEV): 0.70, 1.4, 2.8, 5.6, 11.3, and 22.5% vol/vol. The detailed test method, test concentrations, and calculations for the specific growth rate are found in SI Section 1.5.2.

The EC_50_ for *Daphnia magna* (Crustacean) was determined for samples from the ICEV and BEV using SS-EN ISO 6341:2012. “Determination of the inhibition of the mobility of *Daphnia magna* (Cladocera, Crustacea). Acute toxicity test.” The control samples were tested against K_2_Cr_2_O_7_ and had an average EC_50_, for 24 h of exposure, of 0.9 mg K_2_Cr_2_O_7_ L^–1^, approved range 0.6–2.1 mg L^–1^. For the ICEV water sample, the pH was adjusted to 7.8 ± 0.5 (with NaOH) before testing. The detailed test method and test concentrations are found in SI Section 1.5.3.

## Results and Discussion

### Fire Scenario and Heat-Release Rate

**Figure 1:**
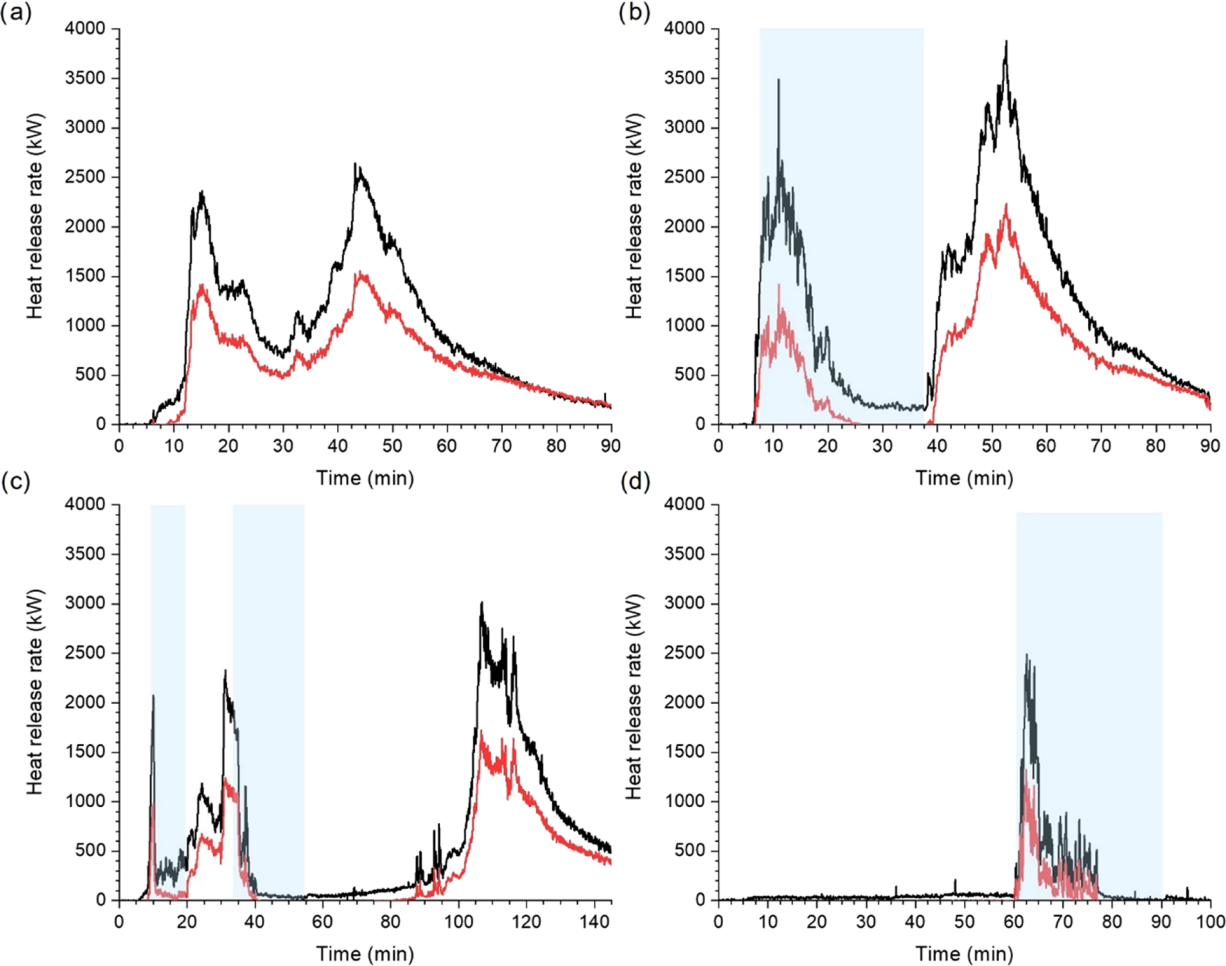
Total (black) and convective (red) HRR for (a) reference test, (b) ICEV, (c) BEV, and (d) battery test. Blue shading indicates the time when the sprinkler system was active.

The detailed timeline, visual observations, and temperature graphs for all tests are presented in SI Table S3 and Figure S3. The HRR was calculated for all four tests, and the graphs are presented in Figure 1. The reference test (vehicle without energy storage) was a free burning test, whilst the remaining three tests were sprinklered tests. The sprinkler system was activated when the HRR reached 1 MW (convective HRR of 668 kW) for the vehicle tests. For the battery test, the sprinkler system was activated 30 s after the visual detection of thermal runaway (HRR of 342 kW, convective HRR of 214 kW).

For the reference test, the HRR graph displayed two peaks. The “split peak” can be explained by the fire propagation. The first peak (*t* = 5–30 min) is from the engine compartment (front of car) burning. At 25 min after ignition of the burner, the left front window collapsed, resulting in ignition of the interior of the cabin. The rear of the vehicle (tires, bumper, etc.) subsequently became involved in the fire, resulting in a second HRR peak (Figure 1a, *t* = 30–90 min). The THR for the vehicle without energy storage was 5.0 GJ. The first peak (engine compartment) contributed to ∼28% of the THR, whereas the second peak contributed to ∼72% of the THR.

For the ICEV test, the sprinkler system was kept active for a pre-set time of 30 min. The HRR continued to increase after activation of the sprinkler system and reached its maximum 2 min and 57 s after activation of the sprinkler system (Figure 1b). The first peak HRR (*t* = 5–30 min) can be assigned to the burning petrol pool and the subsequent rupture of the fuel tank. When the petrol had been combusted, the HRR steadily declined, reaching a “steady state” value of ∼175 kW during the last 10 min with the sprinkler system active. The sprinkler system was turned off at a test time of 37:58. At 45:00, the back windows ruptured and the passenger compartment became involved in the fire, leading to a second HRR peak (*t* = 30–90 min). The THR for the ICEV was 6.1 GJ. The first peak (with the sprinkler system active) contributed to ∼26% of the THR, whereas the second peak contributed to ∼74% of the THR.

For the BEV, the burner was placed beneath the rear part of the battery pack to initiate thermal runaway in the battery as early as possible in the test. The sprinkler system was activated when the HRR reached 1 MW, as in the test with the ICEV. However, upon activation of the sprinkler system, the HRR and battery surface temperatures drastically decreased. One possible reason for the fast-declining HRR could be attributed to the rupture of the rear window. This allowed water to reach the interior of the vehicle, subsequently cooling the top of the battery, as seen by the decreasing battery surface temperature (Δ*T* ∼ 790°) (SI Figure S3d). To avoid using the sprinkler system without having thermal runaway, it was decided to turn off the sprinkler system 10 min after activation. After turning off the sprinkler system, a dry period of 15 min followed when the fire was allowed to grow. A second activation of the sprinkler system was initiated after 15 min, and the sprinkler system was active for an additional 20 min. During the second activation of the sprinkler system, thermal runaway was detected (through visual observation). The HRR graph for the BEV fire test is presented in Figure 1c, and the THR for the BEV was 5.7 GJ. The first two HRR peaks (*t* = 5–55 min, with the sprinkler system active) contributed to ∼27% of the THR, whereas the second peak (*t* = 55–145 min) contributed to ∼73% of the THR.

For the battery pack fire test, the time to initiate thermal runaway was substantially longer than for the BEV. After 60 min of testing, only minor gas venting events had been detected and it was decided to increase the burner power from 30 to 70 kW. The increased burner power immediately triggered thermal runaway in the battery, and the burner power was decreased to 30 kW again. Since the battery pack was shielded from direct impingement of the sprinkler water, no cooling effect of the water on the battery was expected. The HRR graph for the battery test is presented in Figure 1d. The THR for the battery was 0.8 GJ, and the combustion of the battery lasted for 20 min.

**Figure 2:**
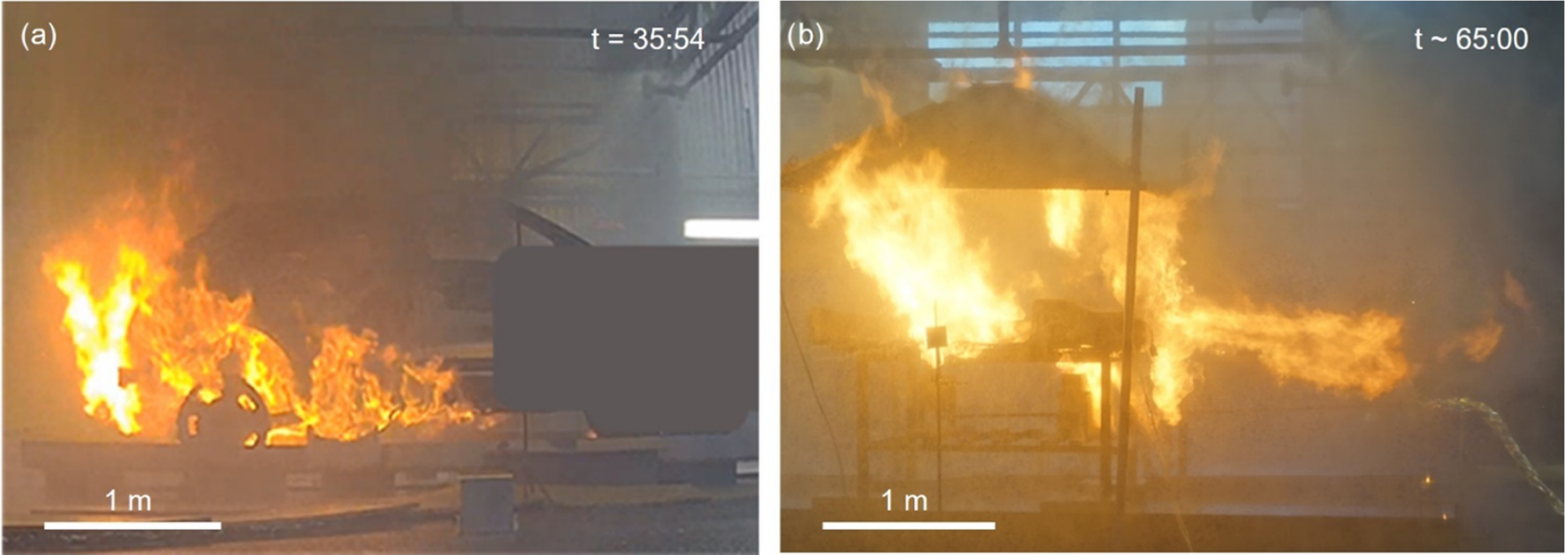
(a) Photograph of thermal runaway during the BEV fire test and (b) photograph of thermal runaway during the battery pack fire test.

The combustion (and venting events) of the free-standing battery was visibly much more intense than for the BEV (Figure 2). One reason for this could be that the gas vents and chassis were efficient in deflecting the jet flames toward the back and below the vehicle. Additionally, the battery was burnt out in 20 min, whilst the BEV fire lasted for more than 140 min.

The total amount of soot was the highest in the reference test, 77 mg m^3^ dry gas, resulting in a total of ∼10 kg of soot (28.8 mg MJ^–1^). For the ICEV, the total amount of soot was 53 mg m^3^ dry gas, resulting in a total of 6.5 kg (19.6 mg MJ^–1^), and the BEV yielded 25.3 mg m^3^ dry gas, resulting in a total of 5.2 kg (9.5 mg MJ^–1^). Considering that during the reference test no energy storage was included, the soot content was decreased by ∼30–70% in the sprinklered tests, indicating that the applied water washed down a large amount of the soot particles.

### Acute Toxicity Tests of Extinguishing Water

The extinguishing water (0–30 min sample) from the ICEV, BEV, and battery fire tests were biologically characterized, and pH, salinity, and conductivity were measured. The results are presented in Table 1. The toxicity was defined by the EC_50_, and the severity criteria of acute toxicity for the evaluated microorganisms can be found in SI Table S4. An EC_50_ below 20% vol/vol was considered to have high toxicity, and an EC_50_ of 20–70% vol/vol was considered to have intermediate toxicity.

**Table 1:**
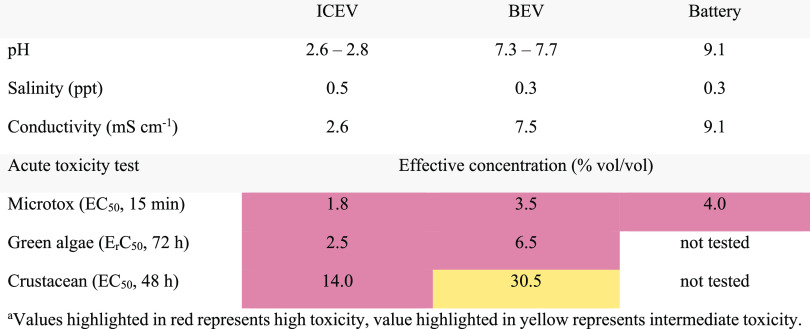
pH, Salinity, Electrical Conductivity, and the EC_50_/E_r_C_50_ for the Tested Species, Measured for Extinguishing Water, 0–30 min Sample*^a^*

In the Microtox analysis (*Vibrio fisheri*), the inhibition of bacteria luminescence was measured. For 15 min of exposure, EC_20_ = 0.35–0.75% vol/vol and EC_50_ = 1.8–4.0% vol/vol, which indicates that all the tested water samples had high toxicity toward *Vibrio fisheri*. The growth inhibition as a function of time and concentration is presented in SI Figure S4.

For green algae (*Pseudokirchneriella subcapitata*), the no-observed-adverse-effect-level (NOAEL) was 0.2 and 0.7% vol/vol (72 h exposure) for the ICEV and BEV, respectively, which indicates that the extinguishing water from both vehicles was highly toxic toward *Pseudokirchneriella subcapitata*. The growth inhibition as a function of concentration and number of cells with respect to time is presented in SI Figure S5.

For crustacean (*Daphnia magna*), the acute toxicity of the ICEV water sample can be described as highly toxic, based on the EC_50_ value being below 20% vol/vol. The water sample from the BEV test showed intermediate toxicity,^35^ as the EC_50_ was 30.5% vol/vol. The NOAEL for 24 h exposure was 3.1 and 25% vol/vol and for 48 h exposure, 3.1 and 12.5% vol/vol for ICEV and BEV, respectively. The number of immobilized *Daphnia magna* for 24 and 48 h for the tested concentrations are presented in SI Table S5.

Interestingly, the pH of the water samples from the BEV and battery tests are remarkably different to the pH of the water from the ICEV test (Table 1). For the BEV test, the water had a pH of 7.3–7.7, which is close to neutral pH of 7.0. The extinguishing water from the battery test was alkaline with a pH of 9.1, whilst the extinguishing water from the ICEV was acidic, with a pH of 2.6–2.8. The reason for the variation in pH has not been further investigated, but the higher pH for the BEV and battery tests could possibly be attributed to the carbonate chemistries found in lithium-ion batteries.^36^ The U.S. Environmental Protection Agency suggests a pH of 6.5–9 as water quality criteria in fresh water.^37^ For many stream species, prolonged durations of a pH below 5 will likely be lethal, resulting in significant changes in species composition and diversity.^38−40^ Short-term exposures of fish to high pH (>9.5) are seldom lethal. However, persistent exposure to pH 9.5–10 can lead to damage to gills, eyes, and skin.^37^ High pH can also contribute to ammonia toxicity^41^ since the ionized form (NH_4_^+^) will form ammonia (NH_3_), as the pH increases.

### Analysis of Metals, Ions, VOCs, PAHs, and PFAS in Extinguishing Water

#### Metals

**Figure 3:**
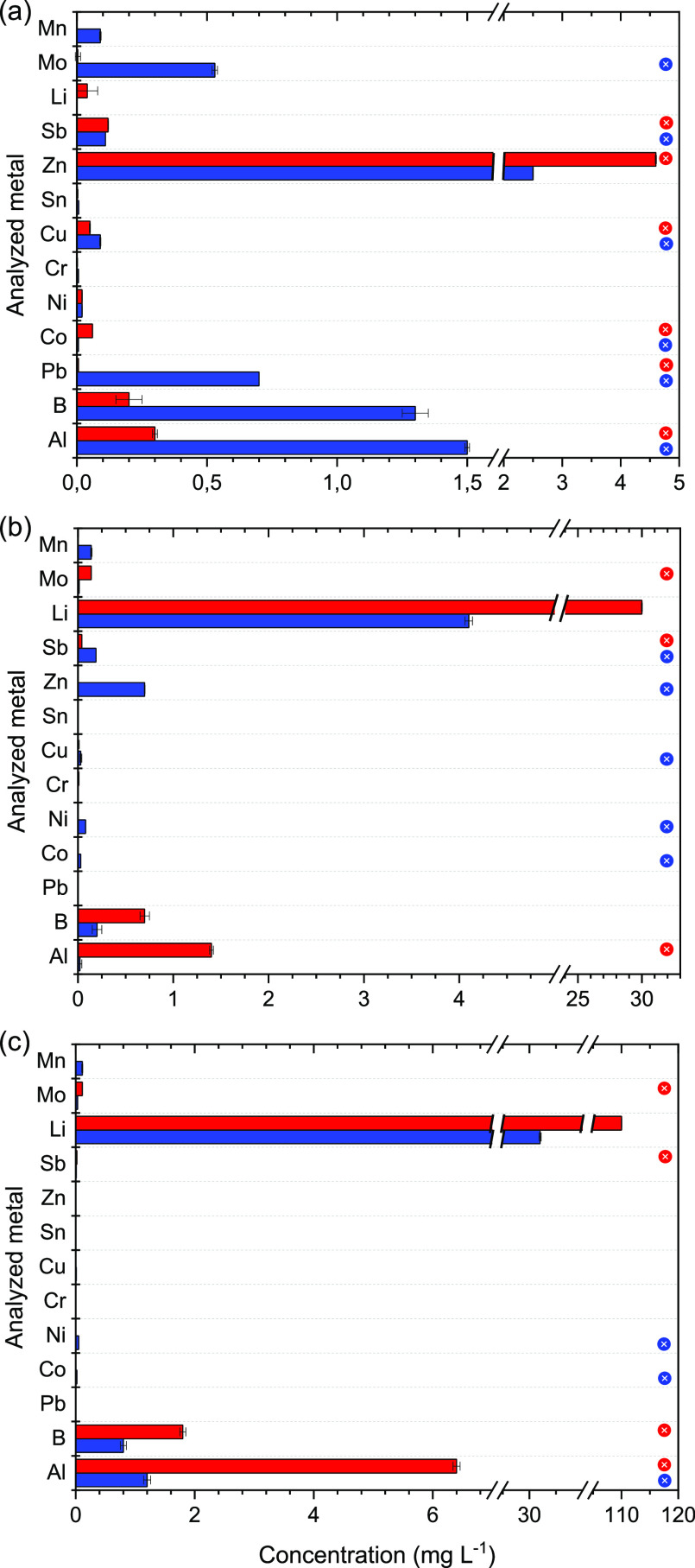
Concentration of metals in extinguishing water from (a) ICEV, (b) BEV, and (c) battery. Pumped water (sample, 0–30 min) is presented in blue, and water taken from the tray after the test is presented in red. The correspondingly colored circles in the right margin indicate that the concentration of the analyzed metal was higher than the surface water guideline value.

Water samples were taken both from the 0–30 min sample (pumped water) and from the tray at the end of each test. Results from the metal analysis of extinguishing water are presented in Figure 3 and SI Table S6. Each analyzed compound was compared to existing surface water guideline values obtained from different regulatory agencies (some of which are found in SI, Table S7). If the concentration of the analyte was higher than the guideline value, it is indicated with a colored dot in the right margin in Figure 3. The guideline value used for comparison was the lower value reported in the SI, Table S7.

Mercury, lead, cadmium, and copper are often highlighted as the more severe environmental pollutants because they are bioaccumulating and have a high toxicity for aquatic organisms. Mercury, cadmium, and arsenic were not found in any of the water samples analyzed in this study. Lead was only found in the water samples collected from the ICEV fire test, at a concentration of 65 μg L^–1^. The recommended surface water guideline value for lead is 30 μg L^–1^.^42^

Copper was found in all tests; the highest concentration of copper was found in the water sample from the ICEV (90 μg L^–1^), the reference test (30 μg L^–1^), and the BEV test (9 μg L^–1^). The surface water guideline value for copper ranges between 9–90 μg L^–1^,^42^ indicating that all analyzed samples were close to or above the guideline value.

The concentration of antimony was high (40–240 μg L^–1^) for all extinguishing water analyzed from the vehicle fire tests compared to the battery test (SI Table S6). Antimony is commonly used in lead-acid batteries to improve the corrosion resistance of the electrodes,^43^ and as a solid lubricant, for example, in the brake pads of vehicles.^43,44^ Antimony as well as chromium, copper, lead, nickel, and zinc are considered priority pollutants by the United States Environmental Protection Agency.^45^

Lithium does not currently have established guideline values in Sweden. However, birth defects have been connected to a high lithium uptake in drinking water (>1 mg L^–1^).^46^ The EC_50_ varies depending on the organisms studied; for *Daphnia magna*, the reported EC_50_ varies between 33–197 mg L^–1^.^47^ The analyzed extinguishing water in this work contained 30 and 110 mg L^–1^ of lithium for the BEV and battery, respectively. Depending on the dilution and site of contamination, this could potentially be harmful to aquatic life and humans.

Battery-specific metals such as manganese, nickel, cobalt, and lithium were found in higher concentrations in the BEV and battery tests compared to the ICEV test. Furthermore, a comparison of metals and ions from the pumped water and the water from the tray after the test showed that most of the analyzed metals were found in higher concentrations in the pumped water. This might be an indication that a vast amount of metal-containing species are washed away with the applied water.

Note that the volume of contaminant and the site of contamination need to be assessed for a holistic view of the severity of pollutants. Some surface water guideline values vary depending on if it is salt or fresh water, as the uptake will be affected by water hardness and pH. Additionally, the sensitivity to each pollutant will differ depending on the recipient, which also needs to be considered. The analyzed compounds presented within this report are only representative for a small number of tests. As the vehicle type, battery chemistry, fire scenario, etc. are varied, the pollutants and concentrations of these will most likely be subjected to variations.

#### Fluoride, Chloride, and Bromide

Fluoride-, chloride-, and bromide-containing compounds were analyzed (Table 2). The surface water guideline values for chloride range between 120–640 mg L^–1^.^48^ The concentrations of chloride in the water samples ranged between 110–1300 mg L^–1^ for the vehicle tests and only 35–50 mg L^–1^ for the battery test. Therefore, most of the chloride can be attributed to the vehicle and not to the traction energy.

**Table 2:** Fluoride, Chloride, and Bromide Concentrations in the Extinguishing Water, Presented in mg L^–1^ (Expected Measurement Uncertainty ∼10%)

		0–30 min sample				sample taken from the tray after the test			
	blank	REF	ICEV	BEV	battery	REF	ICEV	BEV	battery
F^–^	0.15	n.a.	12	4	44	2	8	20	70
Cl^–^	30	n.a.	110	120	35	1300	220	140	50
Br^–^	0.055	n.a.	1	1		38	7	9	4

The fluoride concentrations in the analyzed water samples range between 2–20 mg L^–1^ for the vehicle tests and between 44–70 mg L^–1^ for the battery test. This indicates that most of the fluoride is derived from the battery. Unpolluted fresh water generally has a fluoride concentration of 0.01–0.3 mg L^–1^; for unpolluted seawater, the concentration is somewhat higher, 1.2–1.5 mg L^–1^.^49^ Fluoride can have severe effects on aquatic organisms living in soft waters. In a study by Camargo et al.,^49^ it was suggested that the levels of fluoride ions should be kept below 500 μg L^–1^ to protect the caddisfly larvae (and higher organisms that prey on them) from fluoride pollution. The Canadian Water Quality Guidelines for the Protection of Aquatic Life specify a guideline value of 120 μg L^–1^ of fluoride in fresh water.^50^

#### Time-Resolved Water Sampling of Fluoride

For the BEV and battery fire test, time-resolved water sampling of fluoride was performed. The concentration of fluoride in the blank tests ranged between 0.05–0.3 mg L^–1^. The BEV fire test had a total test time of 150 min, out of these, the sprinkler system was active for 30 min. The concentration of fluoride detected during these 30 min was quite low for the BEV compared to the battery test, 3.5 and 45 mg L^–1^, respectively (see SI Figure S6). However, the time-resolved water sampling did not cover the full BEV fire test, as the sprinkler system was only active for 30 min (20% of the total test time). In comparison to the BEV test, the battery test was substantially shorter. The battery was burnt out in ∼20 min and the sprinkler system was active throughout the test, resulting in a higher concentration of fluoride in the extinguishing water compared to the BEV test. The difference in the end concentration of fluoride for these two tests is most likely an effect of internally flushing the battery pack with water after the test. The internal flushing of the battery was performed to investigate if (or how much) the concentration of contaminants would increase in the extinguishing water upon flushing. The fluoride concentration increased from 20 to 70 mg L^–1^, for the BEV and battery tests, respectively.

#### Volatile Organic Compounds and Polycyclic Aromatic Hydrocarbons

VOCs were only found in the water sample taken after the ICEV fire test, with a total concentration of ∼2600 μg L^–1^. A detailed analysis of the analyzed VOCs is presented in SI Table S8.

Out of the 16 analyzed PAHs, only six PAHs were found in the extinguishing water for the ICEV. For the BEV, only two PAHs were found. The total concentration of PAHs in the water samples was 12.5 and 2.6 μg L^–1^ for the ICEV and BEV tests, respectively. No PAHs were detected in the extinguishing water from the battery fire test. A detailed analysis of the PAHs detected in the extinguishing water can be found in SI Table S9.

**Figure 4:**
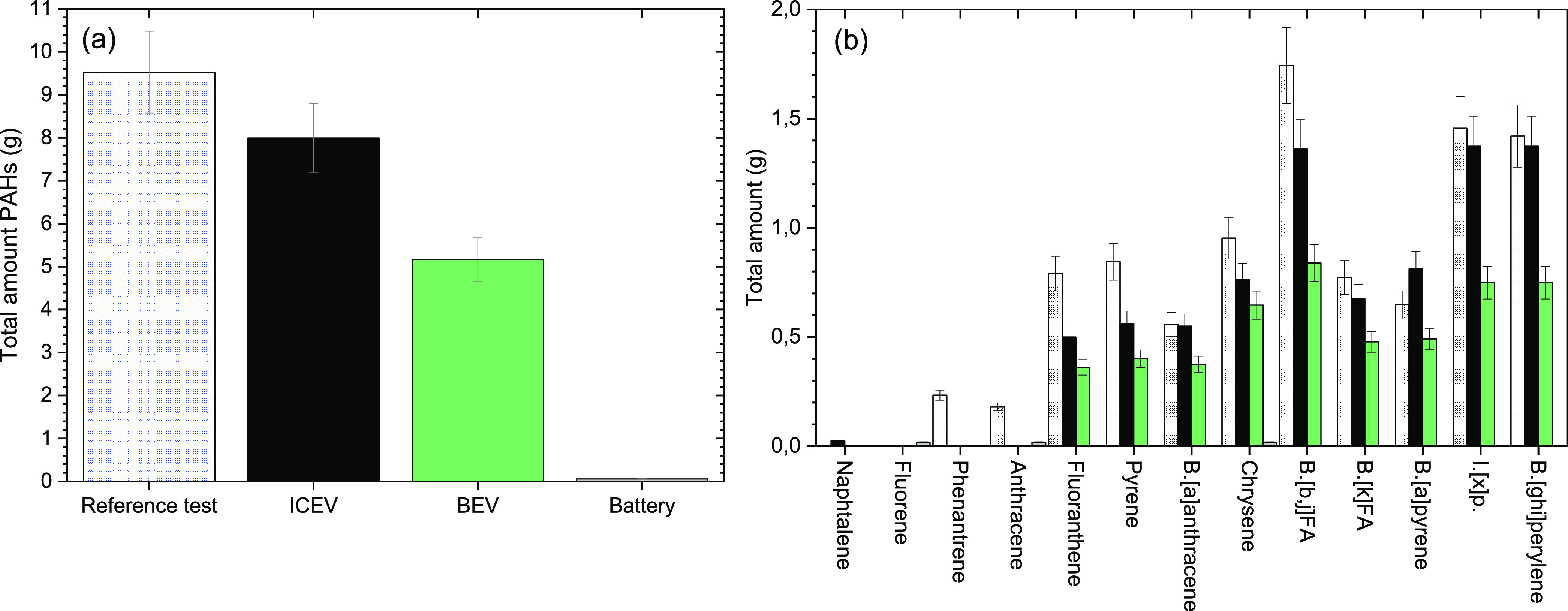
(a) Total amount of particle matter bound PAHs found in the combustion gases for each test. (b) Detailed analysis of each of the 16 PAHs analyzed in the combustion gases. Color coding used for the tests in (a) is also used in (b). Abbreviations used in graph: Benzo (B.), Fluoranthene (FA), and Indeno[1,2,3-cd]pyrene (I.[x]p).

A much higher quantity of PAHs was detected upon analysis of the combustion gases, see Figure 4. These results agree well with previous studies of PAHs in combustion gases compared to extinguishing water.^15^ Acenaphthylene, acenaphthene, and dibenz[*a*,*h*]anthracene were not found in any of the tests performed. The total amount of PAHs found in the vehicle tests was in the range of 5–9.5 g. The highest concentration of PAHs (9.5 g) was found for the reference test (no sprinkler system active). For the battery test, only 3 g of PAHs were detected, indicating that the vehicle itself and the petrol contribute to most of the analyzed PAHs.

#### Per- and Polyfluoroalkyl Substances

**Figure 5:**
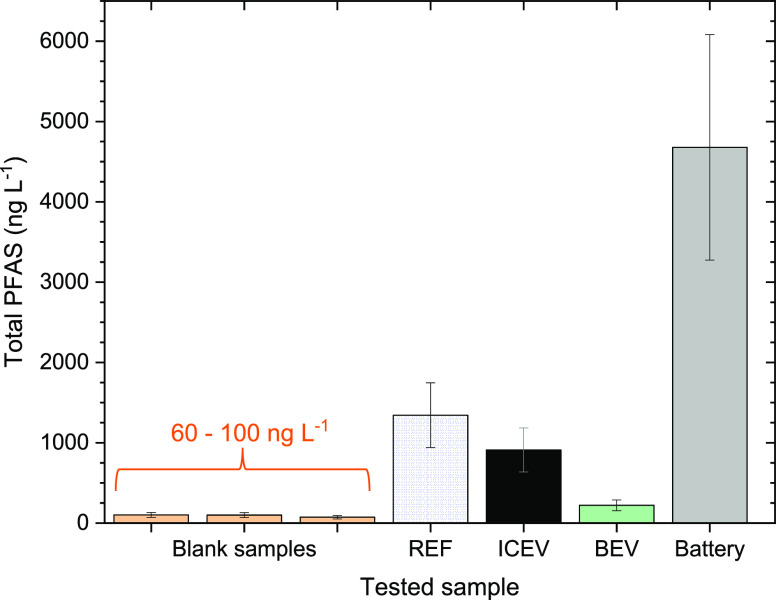
Total concentration of PFAS in the extinguishing water sampled from the tray at the end of each test; blank tests are presented in orange.

PFAS were analyzed for all tests using water samples collected from the tray at the end of each test. To evaluate existing PFAS contamination in the fire laboratory, blank samples were taken before each test. Blank samples indicated that the background concentration of PFAS in the fire laboratory was in the range of 60–100 ng L^–1^. For the blank samples, perfluorohexanoic acid (PFHxA) (60–70 ng L^–1^) was found as the main background contaminant. The detailed analysis of each substance can be found in SI Table S10, and the total concentration of PFAS is presented in Figure 5.

In the reference and ICEV tests, the concentrations of PFAS were similar, indicating that the majority of PFAS derives from the vehicle and not from the petrol. However, for the BEV, the concentration of PFAS was somewhat lower. The reason for this discrepancy is unknown. After the battery test, the pack was opened and flushed with water. Flushing of the battery resulted in a large increase of PFAS in the extinguishing water (Figure 5, SI Table S10). The origin, i.e. the PFAS contributions from the individual components in the battery pack (such as the battery cells and electronics) were not distinguished in this work. The European Commission′s coming limit values for PFAS in drinking water are 500 ng L^–1^ for total PFAS and 100 ng L^–1^ for a sum of 20 PFAS.^51^

PFAS are of a significant environmental concern since they are highly persistent,^52−56^ can bioaccumulate in organisms, and transport through maternal transfer.^57−60^ In work by Sunderland et al.,^61^ the human exposure to PFAS and its epidemiologic evidence for impact on cancer, immune function, metabolic outcomes, and neurodevelopment are reviewed.

Metals, such as lithium, boron, and aluminum (Figure 3 and SI Table S6), were also found in significantly higher concentrations in the water after flushing the battery. Firefighters that respond to BEV fires should consider whether flushing of the battery is necessary, and if it is, where it could be performed in a safe manner to avoid pollution of the environment. Furthermore, extinguishing water from the ICEV fire showed higher toxicity toward the tested aquatic species compared to the extinguishing water collected from the BEV and battery fire. The reason for this could possibly be an effect of the higher concentrations of lead, copper, zinc, VOCs, and PAHs found in the extinguishing water from the ICEV compared to the BEV and battery tests. Nevertheless, to fully assess the severity of pollution, each polluting scenario needs to be assessed individually and the effects of dilution need to be considered. Additionally, results may be subjected to variations depending on the type, size, and model of the tested vehicle.

## Supporting Information Available

Additional experimental details regarding the test setup, sprinkler system, HRR, and acute toxicity tests; additional results regarding the fire tests, chemical analysis, and biological characterization; includes schematic illustrations and photographs of the experimental setup (PDF)
