# Supplementary material for: Ecotoxicity Evaluation of Fire-Extinguishing Water from Large-Scale Battery and Battery Electric Vehicle Fire Tests
Source: Environ Sci Technol. 2023 Mar 13;57(12):4821–30. doi: 10.1021/acs.est.2c08581 (PMC10061927; doi:10.1021/acs.est.2c08581)
Supplement: Supplementary file 1 — es2c08581_si_001.pdf [file es2c08581_si_001.pdf]

Supporting Information

Ecotoxicity evaluation of fire extinguishing water from large-scale battery and battery electric vehicle fire tests

Maria Quant, Ola Willstrand, Tove Mallin, Jonna Hynynen*

RISE Research Institutes of Sweden, Department of Fire and Safety, Brinellgatan 4, 501 15 Borås, Sweden

Contents

Section S1. Material and Methods

Figure S1. An overview of test setup and measurement setup at the large fire hall at RISE, Borås…...4

Section S1.1. Description of the used sprinkler system and water collection system……………4

Figure S2. Photographs of the water collection system……………………………………..5

Section S1.2. Heat release rate calculations…………………………………………………5

Table S1. The 16 external standards used for PAH analysis and results from the blank sample analysis for the combustion gases…...6

Section S1.3. Methods and calculations used for the acute toxicity tests

Section S1.3.1 *Vibrio fischeri*, Microtox…...6

Section S1.3.2 *Pseudokirchneriella subcapitata*, green algae…………………………..6

Section S1.3.3 *Daphnia magna*, crustacean…...7

Section S2. Results and Discussion

Section S2.1. Visual observations for the large-scale fire tests……………………………………8

Table S3. Time of ignition, peak HRR, sprinkler system activation, visual observations regarding energy storage and weight before/after test of vehicles/battery…...8

S2.2 Temperature measurements…………………………………………………………………9

Figure S3. Temperature measured for reference test, battery, ICEV and BEV. Sensor placements are indicated in the schematic on top of each graph, white circle indicates sensor above the propane burner (data for these points are not relevant for comparison and are therefore not shown in graph)……..9

Table S4. Criteria of acute toxicity, EC50……..9

Figure S4. Inhibition of the luminescent bacterium *Vibrio fisheri* after 5, 15 and 30 minutes of incubation in a concentration series of samples a) ICEV 0 – 30 min b) BEV 0 – 30 min and c) battery test 0 – 30 min…………10

Figure S5. Average values of the growth inhibition of Pseudokirchneriella subcapitata after 72 h in a concentration series of ICEV and BEV tests. Photograph from the end of the test and

concentrations tested. The number of cells per ml after 24, 48 and 72 hours for different concentrations of ICEV and BEV water samples……………………………………………10

Table S5. Number of immobilized Daphnia magna for 24 and 48 h for the tested concentrations of extinguishing water from the ICEV and BEV fire test (0 – 30 min sample). As well as the measured pH and oxygen concentration at 0 and 48 h of testing…………………………………11

Table S6. Metal content analysed for the collected water samples, numbers in brackets indicate the standard deviation of the measurement (obtained from the blank sample taken before each test). Values highlighted in yellow indicate that the concentration in the analyzed sample is higher than the corresponding surface water guideline value for that metal. Measurement uncertainty ± 10%...12

Table S7. Surface water guideline values for some of the analyzed compounds in this work. The guideline value used for comparison was the lower value found in table………………………..13

Figure S6. Concentration of fluoride for all time resolved water samples for BEV (green, left y-axis) and battery test (black, right y-axis)………………………………………………………13

Table S8. Concentration of VOCs detected for water sample taken after the ICEV test, the remaining water samples (reference, BEV and battery fire test) were free of VOCs. Limit of quantification 1 µg L^{-1}…………………………………………………………………...14

Table S9. PAH detected in water samples 0 – 30 min and from water taken from the tray at the end of each test. Limit of quantification 0.5 µg L^{-1}……………………………………………14

Table S10. Concentration and type of PFAS for water sample taken after the tests. Limit of quantification, measurement uncertainty ~ 30%...15

References……………………………………………………………………………………16

S1. Material and Methods

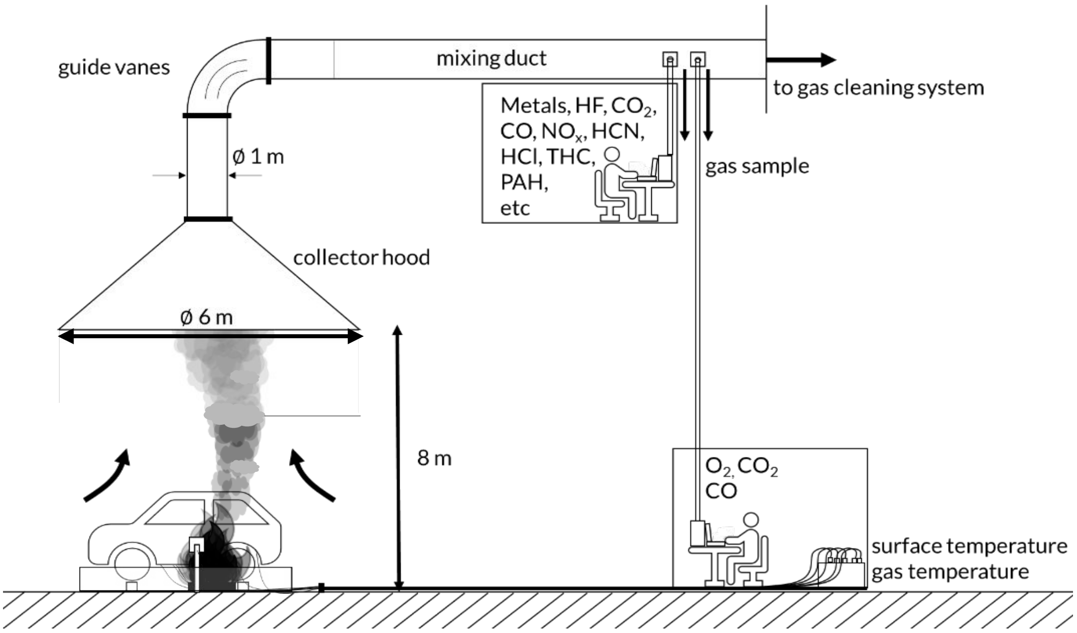

Figure S1. An overview of test setup and measurement setup at the large fire hall at RISE, Borås. Adapted figure from reference [1].

S1.1 Sprinkler system and water collection system

The water discharge density during the tests in this work was set to 10 mm min^{-1}, corresponding to 93 L min^{-1} per sprinkler head, i.e., a total flow rate of 372 L min^{-1}, since four sprinkler heads were used. The sprinkler system was active for 30 min for each test, resulting in 11 160 L of water in total.

Sprinkler heads used were TYCO model Series TY FRB, Quick Response, Standard Coverage sprinklers. The sprinkler heads were fitted with a 3 mm glass bulb, with a nominal operating temperature of 68 °C (the glass bulb was removed for these tests) and had a nominal K-factor of 80.6 $\frac{L\,min^{-1}}{\sqrt{bar}}$. When installed, the plane of the sprinkler frame arms was parallel to the branch lines of the pipe work. The vertical distance between the deflector of the individual sprinkler heads and the bottom of the tray was 2.85 m.

Four sprinkler heads were installed in a hydraulically balanced piping work, having a spacing of 3.05 m by 3.05 m (10 ft. by 10 ft.). Each of the sprinkler heads covered an area of 9.3 m^2. The pipework was constructed from DN50 (2") steel pipe. A plate thermometer was placed in the center of the pipework and a pressure transducer was installed at the end of one of the branch lines. The test object was positioned with its center point between the four sprinklers.

The distribution line of the pipework had a solenoid valve that was remotely operated when the fire reached a convective heat release rate of 667 kW, corresponding to a total heat release rate of 1 MW (estimated roof temperature at 2.85 m of ~ 68°C). For the battery fire test, the activation time was set to 30 s after venting.

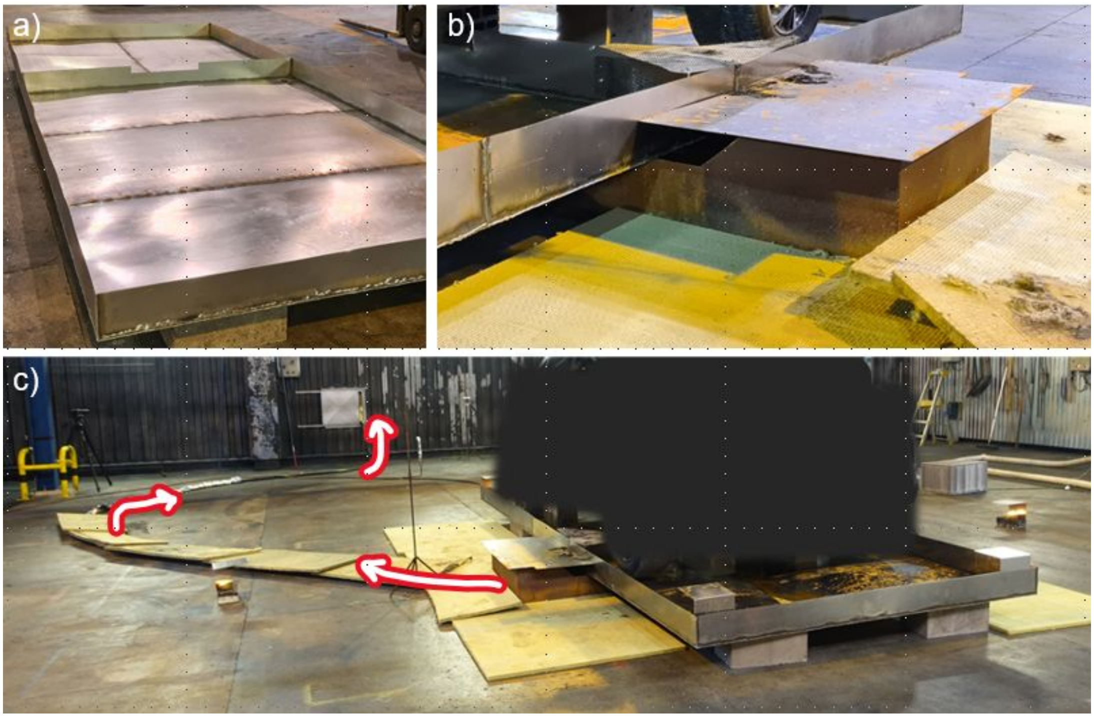

Figure S2. Water collection system, showing a) the large tray (5 x 2 m), b) small pump-tray and c) the complete setup, red arrows indicate the route of the pumped water to the adjacent test hall.

S1.2 Heat release rate calculations

Oxygen, carbon monoxide and carbon dioxide concentrations in the combustion gases as well as mass flow, were measured during the tests. The following equation was applied to calculate the HRR:

$$HRR = E \times \dot{m} \times \frac{M_{O2}}{M_{air}} \times (1 - X^0_{H2O}) \div \left(\frac{\alpha - 1}{X^0_{H2O}} + \frac{1 - \frac{X_{O2}}{1 - X_{CO2}}}{X^0_{O2} - \frac{X_{O2}(1 - X^0_{CO2})}{1 - X_{CO2}}}\right)$$

where E is the energy released per unit mass of O_2 (here 13.1 MJ kg^{-1}), $\dot{m}$ is the mass flow in the exhaust duct (kg s^{-1}), M is the molecular weight, X^0_{H2O}, X^0_{O2}, X^0_{CO2} are the mole fractions of H_2O, O_2 and CO_2 in the incoming air, α is the gas expansion parameter, and X_{O2},X_{CO2} are the mole fractions of O_2 and CO_2 in the exhaust duct.

Table S1. The 16 external standards used for PAH analysis and results from the blank sample analysis for the combustion gas analysis. Limit of quantification 0.1 µg

PAH	Blank sample (µg)
Naphthalene	<0.1
Acenaphthylene	<0.1
Acenaphthene	<0.1
Fluorene	0.2
Phenanthrene	<0.1
Anthracene	<0.1
Fluoranthene	<0.1
Pyrene	<0.1
Benz[a]anthracene	<0.1
Chrysene	<0.1
Benzo[b,j]fluoranthene	0.1
Benzo[k]fluoranthene	0.1
Benzo[a]pyrene	<0.1
Indeno[1,2,3-c,d]pyrene	<0.1
Dibenzo[a,h]anthracene	<0.1
Benzo[g,h,i]perylene	0.1

S1.3 Methods and Calculations for the Acute Toxicity Tests

1.3.1 *Vibrio fischeri,* Microtox

The following equation was applied for calculations of the inhibitory effect on luminescence:

$$H_t = \frac{I_{ct} I_{Tt}}{I_{ct}} * 100$$

Where H_t is the inhibitory effect of the sample after 5, 15 and 30 min of incubation, I_{Tt} is the luminescence at 5, 15 and 30 min of incubation and I_{ct} is the average luminescence from the blanks (5 samples) after 5, 15 and 30 min of incubation.

1.3.2 *Pseudokirchneriella subcapitata,* Green algae

Six replicates were used for controls and triplicates for each test concentration. Algae (100 ml), nutrient medium and sample (extinguishing water) were incubated at room temperature (21 – 24°C) on a rotating shaker with continuous light. pH was measured at the start and after 72 h. Samples were taken from all flasks for cell counting at 24, 48 and 72 h. The following equation was applied to calculate the specific growth rate (µ) for each replicate:

$$\mu = \frac{\ln{(N_L)} - \ln{(N_0)}}{t_L - t_0}$$

where t_0 is the start of testing (days), t_L is the time at which the tests are terminated or the time of the last measurement in the exponential growth period of the control (days), N_0 is the nominal initial cell density and N_L is the nominal cell density at t_L. The mean value of the growth rate (µ) for the control was then calculated.

The growth inhibition (%) in each individual test replicate was then calculated as follows:

$$I_{\mu i} = \left[\frac{\mu_c - \mu_i}{\mu_c}\right] * 100$$

Where $I_{\mu i}$ is the percentage inhibition of growth rate in test replicate i, μ_c is the mean growth rate in the control and μ_i is the growth rate in test replicate *i*.

The inhibition of the control sample mean growth rate was determined and reported as E_rC_{10} and E_rC_{50}. The determination of E_rC_{10} and E_rC_{50} was carried out using graphical interpolation. The lowest ineffective dilution (LID) in the test was considered the zero-effect value (NOEC value). The LID is defined as the highest test concentration where the inhibition is lower than 5%. The requirements for validation of test results according to SS-EN ISO 8692 was met: Growth was exponential, and the cell concentration increased 145-fold over the control in the tests of samples ICEV and BEV. The coefficient of variation in growth rate in the controls sample was 2.7% and pH in the control was changed by 0.7 units.

S1.3.3 *Daphnia magna*, Crustacean

Newly hatched crustaceans, 6 – 24 h old, were incubated for 48 h in a concentration series of the extinguishing water samples collected from the ICEV and BEV fire test. The culture of test species, originated from the Norwegian Institute for Aquatic Research (NIVA), Oslo. As dilution water, aerated M7 medium was used, according to the method OECD TG no.202 (2004). The animals were incubated in 50 ml Petri dishes containing 25 ml of solution. Four replicates with five animals each were used for the control and for each test concentration, test concentrations are presented in Table S2. The dishes were incubated at a temperature of 20 ± 2°C, in dimmed light with a light rhythm of 16 hours light and 8 hours dark. Oxygen concentration and pH were measured in all solutions before the start of each test and after 48 hours. The mobility impairment was determined after 24 and 48 h (Table S5). The water used for dilution had a hardness corresponding to 250 ± 25 mg calcium carbonate per liter and a pH of 7.8 ± 0.5 and was aerated to an initial oxygen saturation of > 80% before use. The test specimens were not fed during the exposure according to the standard protocol SS-EN ISO 6341:2012, "Determination of the inhibition of the mobility of Daphnia magna (Cladocera, Crustacea). Acute toxicity test."

Table S2. Tested concentrations for the water samples (0 – 30 min) for the BEV and ICEV test on acute toxicity test for *Daphnia magna*

Tested water sample	Test concentrations (% v/v)
BEV (0 – 30 min)	3.13, 6.25, 12.5, 25, 50 and 100
Battery (0 – 30 min)	3.13, 6.25, 12.5, 25, 50 and 100

The EC_{50} values, i.e., the concentration at which 50% of the crustaceans were immobilized was determined by graphical interpolation. LID was determined as the highest tested concentration where no more than 10% of crustaceans were immobilized. The requirements for the tests to be compliant with SS-EN ISO 6341:2012 was met: mortality was at most 10% in the control, the sensitivity of the test

system to $K_2Cr_2O_7$ was within the specified range and the content of dissolved oxygen was at least 40% of the saturation value.

2. Results and Discussion

S2.1. Visual Observations

For all tests, ignition of the burner was performed at t = 5 min. Sprinkler system activation, peak HRR and visual observations for each test are summarized in Table S3.

Table S3. Time of ignition, peak HRR, sprinkler system activation, visual observations regarding energy storage and weight before/after test of vehicles/battery

**Reference**		**ICEV**		**BEV**		**Battery**	
**Weight of test object before test (kg)**							
1170		1200		1540		340	
**Time (mm:ss) and observation**							
05:00	Ignition	05:00	Ignition	05:00	Ignition	05:00	Ignition
15:00	First peak HRR	06:21	Pool fire ignited	09:50	Sprinkler activated*	37:55	Puff of white smoke
45:00	Second HRR peak	07:58	Sprinkler activated*	09:00-10:00	TR[1]	60:00	Increase of burner to 70 kW
90:00	Test terminated	10:53	Fuel tank rupture	09:50	Sprinklers activated*[3]	60:00	TR[1,2]
		10:55	1st peak HRR	10:00	1st peak HRR	60:30	Decrease burner 30 kW
		37:58	Sprinklers deactivated	31:20	2cd peak HRR	60:30	Sprinklers activated[4]
		52:36	Second peak HRR	34:50	Sprinklers activated	62:36	Peak HRR
		100:00	Test terminated	36:30	TR[2]	100:00	Test terminated
				56:30	Sprinklers deactivated		
				106:48	3rd peak HRR		
				150:00	Test terminated		
**Weight after test (percentage mass loss)**							
930 kg (20.5%)		891.5 kg (25.7%)		1213 kg (21.2%)		266 kg (21.8%)	
*HRR = 1 MW, [1]Gas temperature above 600°C (battery), [2]Visible signs of TR, [3]active for 10 min, [4]30 s after visible TR active for 30 min							

S2.2 Temperature Measurements

To monitor the temperature development during tests, Type-K thermocouples were placed at different locations on each vehicle and the battery. The location of the thermocouples and temperature graphs are presented in Figure S3.

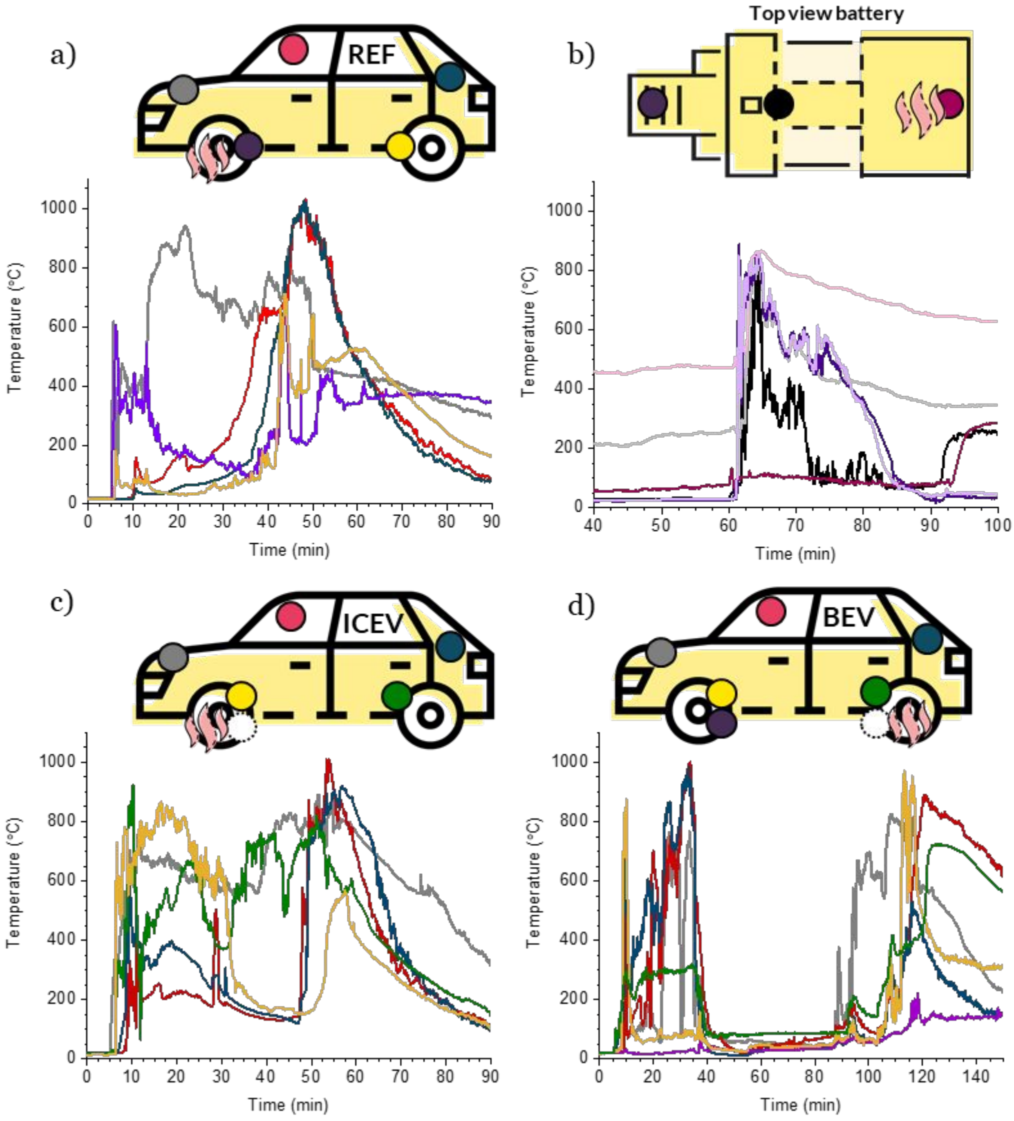

Figure S3. Temperature measured for (a) reference test, (b) battery, (c) ICEV and (d) BEV. Sensor placements are indicated in the schematic on top of each graph, white circle indicates sensor above the propane burner (data for these points are not relevant for comparison and are therefore not shown in graph). Note that the x-scale varies for a-d.

Table S4. Criteria of acute toxicity based on EC_{50} taken from reference [2]

Effective concentration (EC_{50}) (% vol/vol)	Level of toxicity
> 100	Insignificant
70 – 100	Low
20 – 70	Intermediate
< 20	High

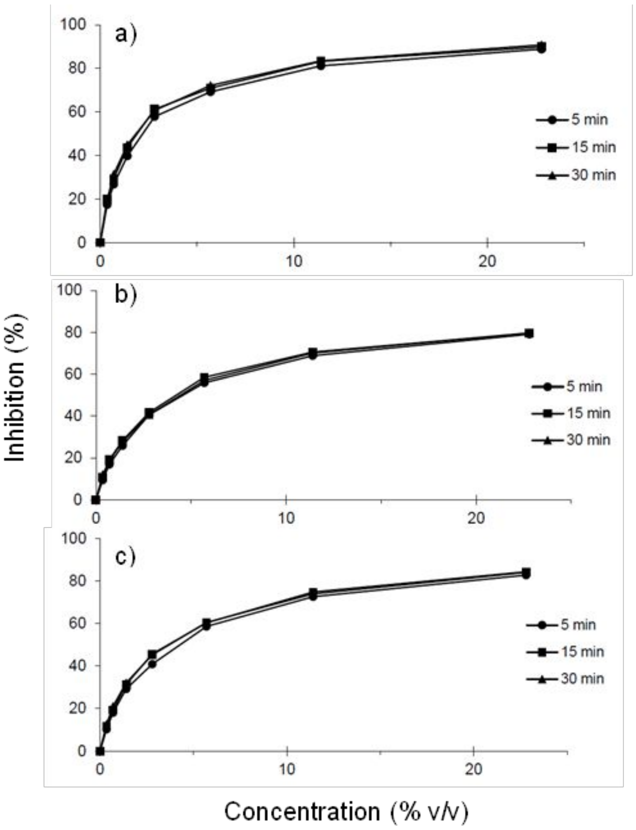

Figure S4. Inhibition of the luminescent bacterium *Vibrio fisheri* after 5, 15 and 30 minutes of incubation in a concentration series of samples a) ICEV 0 – 30 min b) BEV 0 – 30 min and c) battery test 0 – 30 min.

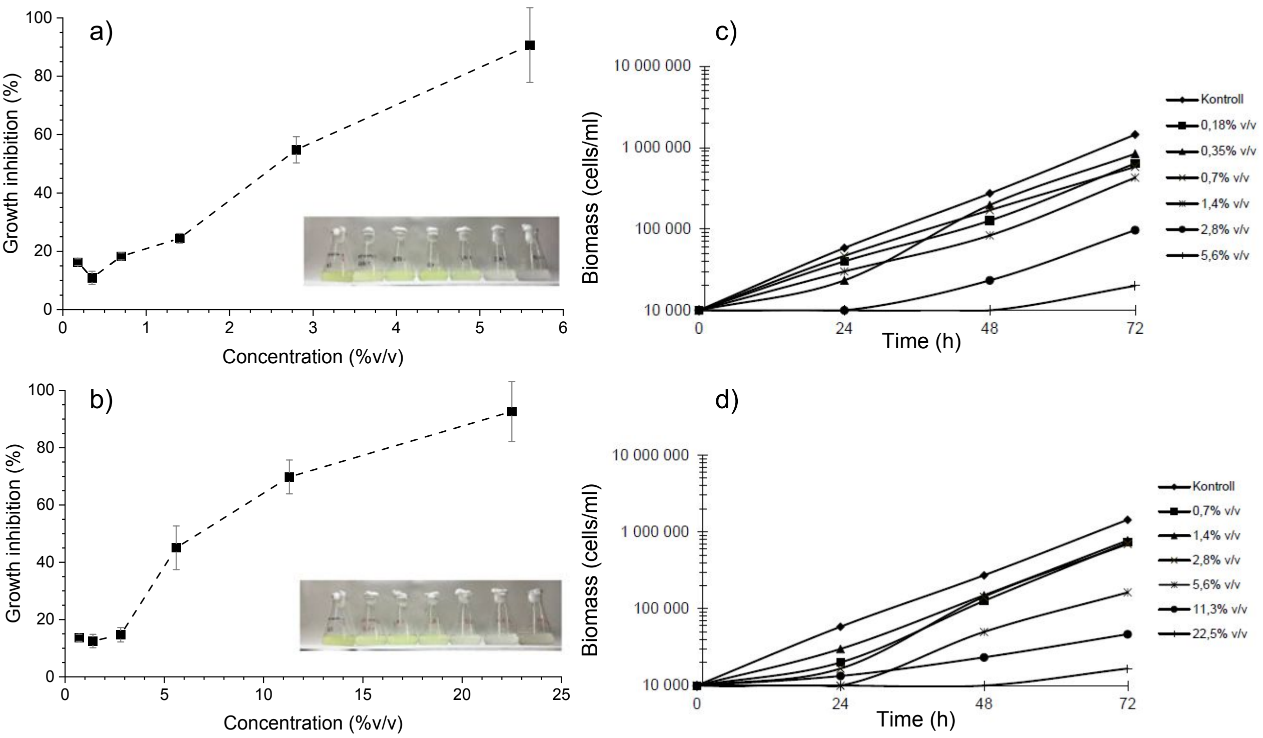

Figure S5. Average values of the growth inhibition of *Pseudokirchneriella subcapitata* after 72 h in a concentration series of sample a) ICEV and b) BEV. Inset in graphs is a photo from the end of the test, the control is on the far left and then progressively higher sample concentration mixture in the right direction. Concentrations tested are presented in (c) and (d) for ICEV and BEV, respectively. The number of cells per ml after 24, 48 and 72 hours for different concentrations of the sample c) ICEV and d) BEV.

Table S5. Number of immobilized *Daphnia magna* for 24 and 48 h for the tested concentrations of extinguishing water run-off from the ICEV and BEV fire test (0 – 30 min sample). As well as the measured pH and oxygen concentration at 0 and 48 h of testing

Tested sample: 0 – 30 min water run-off from ICEV

conc. % v/v	Number of immobilized *Daphnia magna* 24 and 48 h										% immobil.	% immobil.	pH	pH	O_2	O_2
	1		2		3		4		total	total	total	total			mg/l	mg/l
	24	48	24	48	24	48	24	48	24	48	24	48	0h	48h	0h	48h
0	0	0	0	0	0	0	0	0	0	0	**0**	**0**	7,94	7,98	8,83	8,50
3,1	0	1	0	0	0	1	0	0	0	2	**0**	**10**	7,96	7,76	8,96	8,50
6,3	2	2	2	2	1	2	0	1	5	7	**25**	**35**	7,96	7,67	8,90	8,20
12,5	2	2	0	0	3	3	2	3	7	8	**35**	**40**	7,94	7,50	8,93	7,96
25,0	3	5	5	5	3	5	4	5	15	20	**75**	**100**	7,91	7,48	9,00	7,93
50	4	5	5	5	5	5	4	5	18	20	**90**	**100**	7,84	7,41	9,39	7,70
100	5	5	5	5	5	5	5	5	20	20	**100**	**100**	8,21	7,37	10,61	8,08

Tested sample: 0 – 30 min water run-off from BEV

conc. % v/v	Number of immobilized *Daphnia magna* 24 and 48 h										% immobil.	% immobil.	pH	pH	O_2	O_2
	1		2		3		4		total	total	total	total			mg/l	mg/l
	24	48	24	48	24	48	24	48	24	48	24	48	0h	48h	0h	48h
0	0	0	0	0	0	0	0	0	0	0	**0**	**0**	7,94	7,98	8,83	8,50
3,1	0	0	0	0	0	1	0	1	0	2	**0**	**10**	7,96	7,68	8,89	8,35
6,3	0	0	0	0	1	1	0	1	1	2	**5**	**10**	7,99	7,70	8,89	8,35
12,5	0	1	0	0	0	0	0	1	0	2	**0**	**10**	8,04	7,69	8,92	8,34
25,0	0	0	1	2	0	2	1	4	2	8	**10**	**40**	7,92	7,62	8,96	8,23
50	1	3	2	4	2	4	2	4	7	15	**35**	**75**	7,84	7,52	9,14	7,84
100	5	5	5	5	5	5	5	5	20	20	**100**	**100**	7,68	7,57	9,82	8,02

Table S6. Metal content analysed for the collected water samples, numbers in brackets indicate the standard deviation of the measurement (obtained from the blank sample taken before each test). Values highlighted in yellow indicate that the concentration in the analyzed sample is higher than the corresponding surface water guideline value for that metal. Measurement uncertainty ± 10%

	**LOQ§**	**0 – 30 min sample**				**Tray water (taken after test)**			
	**mg L^{-1}**								
		**REF***	**ICEV**	**BEV**	**Battery**	**REF***	**ICEV**	**BEV**	**Battery**
**Al**	<0.0005	n.a	1.5 (0.009)	0.02 (0.02)	1.2 (0.06)	0.01 (0.009)	0.3 (0.009)	1.4 (0.02)	6.4 (0.06)
**B**	<0.05	n.a	1.3 (<0.05)	0.2 (<0.05)	0.8 (<0.05)	0.8 (<0.05)	0.2 (<0.05)	0.7 (<0.05)	1.8 (<0.05)
**Hg**	<0.0005	n.a	-	-	-	-	-	-	-
**Pb**	<0.0001	n.a	0.07 (<0.0005)	-	-	-	0.006 (<0.0001)	-	-
**Cd**	<0.0003	n.a	-	-	-	-	-	-	-
**Co**	<0.0001	n.a	0.006 (<0.0001)	0.03 (<0.0001)	0.02 (0.002)	0.002 (<0.0001)	0.06 (<0.0001)	0.0002 (<0.0001)	-
**Ni**	<0.0005	n.a	0.02 (0.0006)	0.08 (0.0005)	0.05 (0.001)	0.0008 (0.0006)	0.02 (0.0006)	-	-
**Cr**	<0.0003	n.a	0.006 (<0.0003)	-	0.0007 (0.0004)	0.00083 (<0.0003)	0.00035 (<0.0003)	0.01 (<0.0003)	0.0004 (0.0004)
**Cu**	<0.0001	n.a	0.09 (0.0024)	0.03 (0.01)	0.009 (0.002)	0.0036 (0.0024)	0.05 (0.002)	0.003 (0.01)	0.002 (0.002)
**Sn**	<0.0003	n.a	0.007 (<0.0003)	0.0002 (<0.0003)	-	0.0003 (<0.0003)	0.002 (<0.0003)	-	-
**V**	<0.002	n.a	-	-	0.003 (<0.02)	0.006 (<0.002)	-	0.006 (<0.002)	0.004 (<0.02)
**Zn**	<0.002	n.a	2.5	0.7 (0.004)	-	0.004 (0.01	4.6 (0.01)	-	-
**Sb**	<0.0002	n.a	0.11 (0.0012)	0.19 (<0.0002)	0.008 (0.002)	0.24 (0.0012)	0.12 (0.0012)	0.04 (<0.0002)	0.02 (0.002)
**As**	<0.0005	n.a	-	-	-	-	-	-	-
**Li**	<0.04	n.a	-	4.1 (<0.04)	32 (0.2)	0.25 (<0.04)	0.04 (<0.04)	30 (<0.04)	110 (<0.04)
**Mo**	<0.001	n.a	0.53 (0.01)	0.012 (0.0015)	0.03 (0.002)	0.09 (0.01)	0.004 (0.01)	0.14 (0.0015)	0.11 (0.002)
**Mn**	<0.0005	n.a	0.09 (0.003)	0.14 (0.008)	0.11 (0.01)	-	-	-	-

§LOQ = Limit of quantification
(-) indicate that the analysed compound was below the detection limit
*no sprinklers active

Table S7. Surface water guideline values for some of the analyzed compounds in this work. The guideline value used for comparison was the lower value found in table

Substance	Abbreviation	Guideline value (µg L^{-1})	Reference
Aluminum	Al	1 – 4800<br>170	[3]<br>[4]
Boron	B	1500 – 29000	[5]
Chromium	Cr	15 – 150	[6]
Cobalt	Co	4 – 100	[7]
Copper	Cu	9 – 90	[6]
Lithium	Li	2500	[8]
Nickel	Ni	45 – 450	[6]
Manganese	Mn	430 – 3600	[9]
Molybdenum	Mo	73	[10]
Lead	Pb	3 – 30	[6]
Antimony	Sb	10 – 100	[6]
Zinc	Zn	60 – 600	[6]
Chloride	Cl^-	120000 – 640 000	[11]
Fluoride	F^-	120 – 500	[12]
Bromide	Br^-	-	n.a.

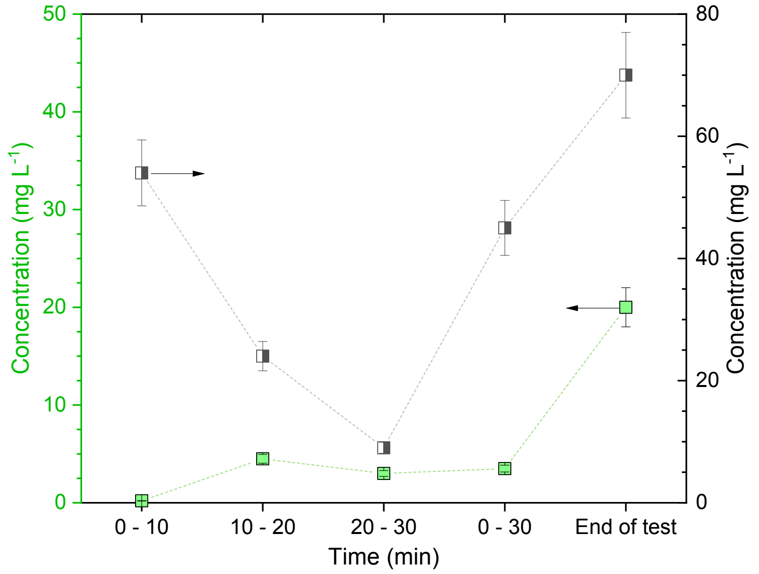

Figure S6. Concentration of fluoride for all time resolved water samples for BEV (green, left y-axis) and battery test (black, right y-axis). Dotted lines are intended as guides for the eye.

Table S8. Concentration of VOCs detected for water sample taken after the ICEV test, the remaining water samples (reference, BEV and battery fire test) were free of VOCs. Limit of quantification 10 µg L^{-1}

**Compound**	**ICEV (µg/L)**
Cyclopentanone	320
2-Propanone, 1-(1-methylethoxy)-	578
Benzonitrile	128
Phenol	367
Ethanone, 2,2-dihydroxy-1-phenyl-	94
2-Acetyl-2-methyltetrahydrofuran	88
Glycidyl isopropyl ether	89
m-Isopropylphenol	65
2-Propenenitrile, 3-phenyl-	68
Caprolactam	391
Phenol, p-tert-butyl-	46
Benzenebutanenitrile	98
Biphenol A	221
unknown	23
**Sum of VOCs**	**2577**
**Internal standards:** DEHP-d4 (deuterated bis(2-ethylhexyl) phthalate<br>**Internal standards Headspace GC-MS:** naphthalene-d8, hexadecane-d34, DEHP-d4	

Table S9. PAH detected in water samples 0 – 30 min and from water taken from the tray at the end of each test. Limit of quantification 0.5 µg L^{-1}

**PAH**	**ICEV T**	**ICEV E**	**BEV T**	**BEV E**	**Battery T**	**Battery E**
(µg L^{-1})						
Naphthalene	1.8	1.8	<0.5	<0.5	<0.5	<0.5
Acenaphthylene	1.0	0.8	<0.5	<0.5	<0.5	<0.5
Acenaphthene	5.0	8.5	1.8	<0.5	<0.5	<0.5
Fluorene	1.3	<0.5	0.8	<0.5	<0.5	<0.5
Phenanthrene	1.7	0.6	<0.5	<0.5	<0.5	<0.5
Anthracene	1.5	1.0	<0.5	<0.5	<0.5	<0.5
**Sum of 16 PAHs**	**12.3**	**12.7**	**2.6**	**-**	**-**	**-**
**(–)** below detection limit<br>**T** – time resolved testing (time 0 – 30 min)<br>**E** – end of test						

Table S10. Limit of quantification and concentration of targeted PFAS for blank samples and water samples taken after the tests, measurement uncertainty ± 30%

PFAS	LOQ	Blank 1	Blank 2	Blank 3	Reference	ICEV	BEV	Battery
ng L^{-1} (ppt)								
PFBA	50	-	-	-	-	46	-	113
PFPA	50	-	-	-	68	137	-	101
PFBS	10	-	-	-	97	-	137	2252
PFHxA	10	71	60	62	113	215	-	268
PFPS	10	-	-	-	-	-	-	-
PFHpA	10	12	-	-	-	24	-	66
PFHxS	10	-	-	-	-	-	-	64
PFOA	10	-	-	-	12	19	12	139
6:2 FTS	10	-	32	-	1019	447	47	1313
PFHpS	10	-	-	-	-	-	-	-
PFNA	10	-	-	-	-	-	-	-
PFOS	10	-	-	-	-	-	-	348
PFDA	10	-	-	-	-	-	-	-
PFNS	10	-	-	-	-	-	-	-
PFUdA	10	-	-	-	-	-	-	-
PFDS	10	-	-	-	-	-	-	-
PFDoDA	10	-	-	-	-	-	-	-
PFUdS	10	-	-	-	-	-	-	-
PFTrDA	10	-	-	-	-	-	-	-
PFDoDS	10	-	-	-	-	-	-	-
PFTeDA	10	-	-	-	-	-	-	-
PFTrDS	10	-	-	-	-	14	-	-
**Sum PFAS**		**83**	**92**	**62**	**1309**	**888**	**196**	**4664**

PFAS abbreviations

PFBA - perfluorobutanoic acid
PFPeA - perfluoropentanoic acid
PFBS - perfluorobutanesulfonic acid
PFHxA - perfluorohexanoic acid
PFPS - perfluoropentanesulfonic acid
PFHpA - perfluoroheptanoic acid
PFHxS - perfluorohexanesulfonic acid
PFOA - perfluorooctanoic acid
6:2 FTS - 6:2 fluorotelomer sulfonic acid
PFHpS - perfluoroheptanesulfonic acid
PFNA - perfluorononanoic acid
PFOS - perfluorooctane sulfonate
PFDA - perfluorodecanoic acid
PFNS - perfluorononanesulfonic acid
PFUdA - perfluoroundecanoic acid
PFDS - perfluorodecane sulfonic acid
PFDoDA - perfluorododecanoic acid
PFUdS - perfluoroundecanesulfonic acid
PFTrDA - perfluorotridecanoic acid
PFDoDS - perfluorododecane sulfonic acid
PFTeDA - perfluorotetradecanoic acid
PFTrDS – perfluorotetradecanesulfonic acid

References

(1) Willstrand, O.; Bisschop, R.; Temple, A.; Anderson, J. *Toxic Gases from Fire in Electric Vehicles*; 2020.

(2) Naturvårdsverket. *Handbok 2010:3 Kemisk Och Biologisk Karakterisering Av Punktutsläpp till Vatten*; 2011.

(3) EPA. *Aquatic Life Ambient Water Quality Criteria for Aluminum in Freshwater*; Washington, 2018.

(4) *Canadian Environmental Protection Act, 1999 - Federal environmental quality guidelines - aluminium*. https://www.canada.ca/en/environment-climate-change/services/evaluating-existing-substances/federal-environmental-quality-guidelines-aluminium.html (accessed 2022-04-06).

(5) CCME. *Canadian water quality guidelines for the protection of aquatic life: Boron*. https://www.ccme.ca/en/res/boron-en-canadian-water-quality-guidelines-for-the-protection-of-aquatic-life.pdf (accessed 2022-04-06).

(6) Naturvårdsverket. *Metodik För Inventering Av Förorenade Områden*; 1999.

(7) Nagpal, N. K. *Technical Report - Water Quality Guidelines for Cobalt*; Victoria BC, 2004.

(8) CCME. *Water quality guidelines for the protection of agriculture: Lithium*. https://www.ccme.ca/en/chemical/127#_ag_irrigation_concentration (accessed 2022-04-06).

(9) CCME. *Canadian water quality guidelines for the protection of aquatic life: Manganese*. https://www.ccme.ca/en/res/manganese-en-canadian-water-quality-guidelines-for-the-protection-of-aquatic-life.pdf (accessed 2022-04-06).

(10) CCME. *Canadian water quality guidelines for the protection of aquatic life: Molybdenum*. https://www.ccme.ca/en/res/molybdenum-en-canadian-water-quality-guidelines-for-the-protection-of-aquatic-life.pdf (accessed 2022-04-06).

(11) CCME. *Canadian water quality guidelines for the protection of aquatic life: Chloride*. https://www.ccme.ca/en/res/chloride-en-canadian-water-quality-guidelines-for-the-protection-of-aquatic-life.pdf (accessed 2022-04-06).

(12) CCME. *Canadian water quality guidelines for the protection of aquatic life: Inorganic Fluorides*. https://www.ccme.ca/en/res/fluorides-inorganic-en-canadian-water-quality-guidelines-for-the-protection-of-aquatic-life.pdf (accessed 2022-04-06).
